# Climate Change and Emergency Medicine: A Scoping Review Across Emergency Medicine Subspecialties

**DOI:** 10.5811/westjem.52979

**Published:** 2026-05-13

**Authors:** Lea Moujaes, Kayla Iuliucci, Stefan Wheat

**Affiliations:** * Johns Hopkins University School of Medicine, Department of Emergency Medicine, Baltimore, Maryland; † University of Washington, Department of Emergency Medicine, Seattle, Washington

## Abstract

**Introduction:**

Climate change is reshaping emergency medicine (EM) practice through rising temperatures, extreme weather events, and deteriorating air quality. Emergency medicine serves as a critical frontline indicator for climate-sensitive health conditions, yet evidence describing climate impacts across EM subspecialties remains fragmented. This scoping review synthesizes existing literature at the intersection of climate change and EM to identify key findings, knowledge gaps, and priorities for building climate-resilient emergency care systems.

**Methods:**

We conducted a scoping review with reporting aligned to the PRISMA Extension for Scoping Reviews. We searched PubMed, Scopus, and Embase through March 2025, combining climate-related terms with EM terms. Two independent reviewers screened 794 articles, with 35 studies meeting inclusion criteria. We extracted data on study characteristics, climate exposures, EM outcomes, vulnerable populations, and system-level impacts across five EM subspecialties: emergency medical services; trauma; disaster medicine; toxicology; and mental health.

**Results:**

Across 35 studies spanning five EM subspecialties, most examined temperature-related exposures, with additional focus on extreme weather events and air quality. In emergency medical services, heatwaves and compound climate events were associated with increased call volume and operational strain, with vulnerabilities identified among older adults, working-age males, and populations in resource-limited settings. Trauma studies demonstrated consistent associations between ambient temperature and injury patterns, including traffic injuries, falls, and assaults with reproducible lag effects of 1–6 days. Disaster medicine studies highlighted critical preparedness and infrastructure gaps, including limited emergency management capacity, and predictable post-event surges in emergency department (ED) utilization. Toxicology studies linked higher temperatures and air quality changes to increased emergency visits for substance-related overdoses and respiratory conditions, while mental health studies consistently reported increased ED use and hospitalizations for psychiatric and substance use disorders during periods of extreme heat. Across subspecialties, socially marginalized populations, including individuals experiencing homelessness, those of lower socioeconomic status, older adults, and people with mental health or substance use disorders were disproportionately affected.

**Conclusion:**

Climate change is placing increasing strain on emergency care systems while amplifying existing health inequities. Current evidence is limited by geographic concentration in high-income settings, a predominant focus on temperature-related hazards, a lack of evaluated interventions, and insufficient integration of climate projections into health system planning. Addressing these gaps will be essential for developing climate-informed emergency medicine strategies capable of protecting vulnerable populations as climate-related health risks intensify.

## INTRODUCTION

Climate change represents a critical population health emergency, fundamentally altering disease patterns and healthcare utilization while disproportionately impacting vulnerable communities.^1^,^2^ Rising global temperatures, increased frequency of extreme weather events, and deteriorating air quality are creating unprecedented challenges for healthcare systems worldwide, with emergency medicine (EM) at the frontline for climate-related health impacts among the most at-risk populations.^3^,^4^

Emergency departments and emergency medical services (EMS) function as critical safety nets for climate-sensitive health conditions, including heat emergencies, respiratory exacerbations, mental health concerns, and increased incidence of trauma. These climate-related presentations strain emergency care systems while highlighting the uneven distribution of climate health burdens across different populations and geographic regions.^4^,^5^ Beyond direct patient care, climate change poses operational challenges that threaten the delivery of equitable emergency care. Extreme weather events can overwhelm emergency services in vulnerable communities, disrupt transportation networks essential for accessing care, and damage healthcare infrastructure in areas with limited adaptive capacity.^6^,^7^ These cascading effects underscore the urgent need for resilient, climate-informed emergency care systems capable of protecting the most vulnerable during climate emergencies.^8^,^9^

While individual studies have documented associations between weather patterns and specific health outcomes,^10^–^14^ comprehensive understanding of climate impacts across EM subspecialties including EMS, trauma, toxicology, disaster medicine, and mental health remains fragmented, particularly among high-risk communities. Current EM training curricula provide limited guidance on climate-informed care, leaving practitioners underprepared to respond effectively to the growing frequency and intensity of climate-related disasters.^15^–^17^

This scoping review maps existing literature at the intersection of climate change and EM, identifying key findings, knowledge gaps, and educational needs across subspecialties with particular attention to vulnerable populations. By synthesizing evidence on climate exposures and emergency healthcare use, this review will inform development of climate-informed emergency care strategies essential for building resilient, equitable EM systems capable of effectively serving vulnerable communities facing accelerating climate impacts.

## METHODS

### Study Design

We conducted a scoping review to characterize existing literature at the intersection of climate change and EM across five subspecialties: EMS; trauma; disaster medicine; toxicology; and mental health. This scoping review was conducted to map the breadth of evidence and identify knowledge gaps across heterogeneous exposures, outcomes and study designs. Reporting of this review aligns with the Preferred Reporting Items for Systematic Reviews and Meta-Analyses–Extension for Scoping Reviews.

**Population Health Research Capsule**

What do we already know about this issue?

*Climate change increases ED visits, EMS demand, and worsens outcomes, disproportionately affecting vulnerable populations*.

What was the research question?

*What are climate change impacts, gaps, and priorities across EM subspecialties?*

What was the major finding of the study?

*Across 35 studies, heat exposure was associated with increased injury risk (RR 1.08/°C, 95% CI 1.03–1.14) and higher emergency care utilization*.

How does this improve population health?

*We identify urgent gaps to guide climate-resilient emergency systems and mitigate escalating, inequitable climate health impacts*.

### Search Strategy

We conducted comprehensive searches in PubMed, Scopus, and Embase through March 2025 in collaboration with a medical librarian. We used controlled vocabulary (MeSH, Emtree) and free-text terms combining climate-related terms (“climate change,” “global warming,” “extreme weather,” “heat wave”) with EM terms (“emergency medicine,” “emergency department,” “emergency medical services”). Google Scholar was searched for additional gray literature. Gray literature was defined as non–peer-reviewed scholarly content indexed within Google Scholar, such as reports, theses, or conference proceedings. We conducted searches using predefined strategy and fixed time window; studies published after completion of the initial search were not eligible for inclusion.

## ELIGIBILITY CRITERIA

Inclusion criteria were as follows: articles discussing climate change or environmental exposures/events related to EM subspecialties, published in English, describing empirical research, interventions, or conceptual frameworks. Exclusion criteria were articles not addressing EM contexts or consisting solely of editorials without empirical content.

### Study Selection

After duplicate removal, 794 articles underwent title/abstract screening by two independent reviewers. Articles meeting criteria advanced to full-text review, with 35 meeting final inclusion criteria.

### Data Extraction

Two reviewers independently extracted data using a standardized abstraction framework, with discrepancies resolved through discussion. Extracted elements included study characteristics (design, location, population, subspecialty); climate exposures (temperature, extreme weather, air quality); EM outcomes (ED visits, EMS calls, hospitalizations), vulnerable populations and temporal patterns, and system-level impacts, including educational or operational considerations.

## RESULTS

Thirty-five studies spanning five EM subspecialties met inclusion criteria: EMS (n = 13); trauma (n = 4); disaster medicine (n = 7); toxicology (n = 2); and mental health (n = 9). Studies were conducted primarily in Asia, Europe, North America, and Australia, with limited representation from low-resource regions. Observational designs predominated, including time-series analyses, retrospective ecological studies, case–crossover designs, Delphi methods, and qualitative approaches (Table 1).

### Emergency Medical Services

Most studies focused on temperature-related exposures, particularly heat waves and ambient temperature effects on EMS demand. In Japan, analysis of heat stroke–related ambulance transports across all 47 prefectures using wet-bulb globe temperature showed regional variation in risk, with evidence of heat adaptation in chronically warmer areas.^18^ In Queensland, Australia, ambulance calls increased by 12.68% during heatwaves, with the largest proportional increases occurring during low- and severe-intensity events and among residents of very remote areas and major cities.^19^ Injury-related EMS demand was also climate-sensitive. In Chengdu, China, temperatures above 17.9 °C were associated with higher injury risk (risk ratio [RR] 1.08 per 1 °C increase, 95% CI, 1.03–1.14), peaking at 2–4 days.^20^ In Shenzhen, China, compound cold and strong monsoon events, and periods of low temperatures accompanied by heavy precipitation, were associated with increased all-cause calls (cumulative RR [CRR] 1.401) and endocrine-related calls (CRR 1.641), while compound heat waves combined with lightning significantly impacted digestive and endocrine disease presentations, as reported in observational analyses.^21^

Consensus-building studies, including Delphi methods that synthesize expert agreement across multiple rounds of structure surveys, further underscored systemic vulnerabilities. A Finnish Delphi study with EMS and emergency department experts identified 14 climate-related challenges across health impacts, workload, operational strain, and societal disruption.^6^ An international Delphi study with experts from multiple countries highlighted 18 consensus statements on heat wave–related overload, underscoring disparities between the income level of countries and the need for system-level adaptation planning.^22^

### Trauma

Quantitative analyses consistently linked extreme temperatures to trauma-related emergencies. In Chongqing, China, high temperatures (32 °C vs 9 °C) increased risks of traffic accidents (CRR = 1.346, 95% CI, 1.167–1.552), assault-related injuries (CRR = 1.508), falls from height (CRR = 1.871), and sharp injuries (CRR = 2.112), while low temperatures (7 °C) increased fall risk (CRR = 1.220).^23^ In Chengdu, temperatures above 17.9 °C raised injury risk by 1.08-fold per 1 °C (95% CI, 1.03–1.14), with traffic accidents, poisoning, and falls most common.^20^ In Korea, nontraumatic injuries, defined as injuries such as drowning, poisoning, burns, electrical injuries, and other non-mechanical injury mechanisms, increased by 1.95% per °C (95% CI. 1.28–2.62%), while traumatic injuries followed nonlinear patterns with a threshold around 0 °C.^24^

Across studies, consistent lag effects (1–6 days) and heightened risks among males and adults were observed. A policy analysis from Pakistan emphasized inadequate health expenditure, rural–urban disparities, and climate-related trauma burdens. 25

### Disaster Medicine

Studies consistently revealed infrastructure and preparedness gaps. In China, only 36.5% of hospitals maintained emergency management offices, while 74% of nurses reported inadequate heat-preparedness knowledge.^26^ In Pakistan, limited health expenditure and rural–urban disparities constrained capacity.^25^ Temporal analyses showed rising disaster frequency. In Taiwan, extreme weather accounted for 92.2% of events reported to regional emergency medical operation centers (2014–2018).^27^ A global review reported a fivefold increase in major climate-related disasters over 50 years.^28^ In Ireland’s 2018 blizzard, emergency department volumes initially decreased during snowfall but surged afterward, with 71 cases distributed across injuries, medical, logistical, and social presentations.^29^

Operational adaptations, such as reallocation of emergency department and inpatient beds, workflow changes to maintain patient throughput, and flexible staffing and transfer practices implemented during disaster response, were also observed. During Hurricane Harvey, despite 74% inpatient bed loss, median emergency department length of stay decreased for admitted patients (591 vs 723 minutes) and discharges (261 vs 336 minutes), reflecting changes in care delivery processes implemented during disaster response rather than demonstrated causal effects.^30^

### Toxicology

Two United States (U.S.) studies linked climate exposures to toxicologic emergencies. In California, analysis of more than 3.4 million ED visits showed higher daily temperatures associated with overdoses, compared with non-overdose ED visits, particularly amphetamines (odds ratio 1.15, 95% CI, 1.09–1.22 comparing 95^th^ vs 50^th^ percentile), as well as cocaine and opioids. Effects were strongest for overdoses.^31^ A projection study estimated that under Representative Concentration Pathway (RCP) 4.5, a moderate emissions scenario, compared to RCP 8.5, a high emissions scenario, approximately 3,100 asthma ED visits and $1.7M in costs could be averted, underscoring benefits of climate mitigation.^32^

### Mental Health

All included studies reported positive associations between elevated temperatures and mental health–related ED use or hospitalizations.^33^–^40^ In Brazil, lower socioeconomic status public patients were more vulnerable than private patients.^36^ In Beijing, increased projected burdens, defined as higher ED visits and hospital admissions, were observed for schizophrenia, substance-use disorders, women, and adults < 65 years of age.^38^ In Toronto, extreme heat increased ED visits for schizophrenia, mood, and neurotic disorders by 29% (99^th^ vs 50^th^ percentile).^39^

In the U.S., heat was linked to 3–4 fold increases in admissions for depression, schizophrenia, and bipolar disorder, based on nationwide time-stratified case–crossover analyses comparing exposure on admission days with matched control days^33^; and other national analyses documented increased ED visits and suicide risk during heat exposure.^35^,^37^,^38^ Canadian studies identified increased ED visits counts and hospital admissions for schizophrenia, dementia, and substance misuse during periods of elevated temperature.^34^ In Sydney, Australia, homeless men were identified as particularly vulnerable to heat-related illness with significant healthcare costs.^40^

Across subspecialties, several consistent patterns of vulnerabiliy emerged. Males and working-age adults faced higher risks in trauma and EMS studies, while older adults, individuals with substance use or psychiatric disorders, and socially marginalized populations such as individuals experiencing homelessness were disproportionately affected across multiple domains. Systems-level gaps in preparedness, resource allocation, and climate adaptation were identified in multiple studies. These vulnerabilities and system-level findings are summarized in Table 2.

## DISCUSSION

### Emergency Medical Services

Climate change is fundamentally altering the landscape of EMS through multiple interconnected pathways that extend beyond direct heat-related health impacts. By modifying environmental conditions, extreme weather patterns, and population vulnerability profiles, climate change is creating unprecedented challenges for prehospital emergency care systems worldwide.^6^ In this scoping review, we identified 13 papers examining associations between climate-related exposures and EMS use across diverse geographic contexts. Collectively, these studies demonstrate that temperature extremes and compound weather events are consistently associated with increased EMS demand, underscoring EMS as a critical frontline indicator for emerging climate-sensitive health risks.^18^,^19^,^21^

The predominant focus on temperature-related exposures reflects both the relative maturity of heat-health research and the immediate operational challenges posed by extreme heat events to emergency medical systems. Several large-scale analyses demonstrated graded, nonlinear, or threshold-based associations between ambient temperature and EMS utilization, with evidence suggesting partial regional adaptation in chronically warmer settings.^18^ However, elevated risks persisted during extreme heat events, particularly among older adults, indicating that adaptation remains incomplete and unevenly distributed.^18^,^19^ Studies examining compound weather exposures suggest that simultaneous hazards, such as low temperature combined with heavy precipitation, may amplify EMS demand beyond what is captured by single-exposure frameworks.^21^ These findings highlight the importance of moving beyond isolated climate variables toward more integrated exposure models that better reflect real-word conditions.^21^

Across EMS literature, several limitations were consistent, including reliance on ecological (population-level) study designs, limited representation of low-resource settings, and minimal integration of climate projections. These gaps constrain the ability of EMS systems to anticipate future demand and to design proactive, climate-informed response strategies, reinforcing the need for broader geographic coverage and forward-looking analysis.^22^,^41^

### Trauma

Building on the evidence that heat contributes to surges in EMS demand, we identified four studies examining association between temperature and trauma-related emergency care utilization. While multiple environmental pathways may influence injury risk, including occupational exposures, behavioral changes, and infrastructure vulnerabilities, the four studies reviewed here focus specifically on temperature as a primary exposure of interest. Together, they demonstrate that fluctuations in temperature, particularly extreme heat, are associated with consistent patterns of trauma-related demand for EMS and ED use across diverse geographic and socioeconomic settings.^20^,^23^–^25^

Across studies conducted in China and South Korea, increases in temperature were associated with higher volumes of trauma-related emergency presentations across multiple injury categories, including traffic accidents and falls. 20,23 Several analyses reported nonlinear or threshold-based associations between temperature and injury-related utilization;^23^,^24^ however, given the observational nature of the included studies, the available evidence does not permit determination of causal mechanisms or formal dose–response relationships.

Despite differences in climate, methodology, and population demographics, males and young to middle-aged adults were consistently identified as groups experiencing higher temperature-related trauma burden. These patterns may reflect occupational exposures, behavioral changes, or increased outdoor activity during warmer conditions. However, all included studies were observational, limiting causal inference.^20^,^23^–^25^ Notably, none of the included trauma studies evaluated preventive or adaptive interventions such as responder training, early warning systems, or public education strategies, despite the predictability of temperature-related injury patterns. This absence highlights a translational gap between epidemiologic evidence and actionable trauma prevention strategies, a theme echoed across other EM subspecialties and further detailed in the Common Gaps and Priorities section.

### Disaster Medicine

Beyond injuries linked to heat and behavior, climate change also magnifies large-scale disasters that disrupt entire emergency care systems. Seven studies reviewed here showed that climate-related events consistently produced predictable surges in healthcare utilization, exposed systemic weaknesses, and highlighted opportunities for adaptation.^26^,^27^,^29^,^30^ Common vulnerabilities emerged across diverse hazards and geographies: inadequate infrastructure; rural–urban disparities; insufficient staffing; and gaps between perceived and actual preparedness. Several studies documented predictable temporal patterns of healthcare demand surrounding disaster events, yet none evaluated systematic educational interventions or formal clinician training, leaving a critical translational gap between surveillance and actionable preparedness.

Equity dimensions were particularly pronounced. High-income countries leveraged sophisticated data systems and analytic approaches to quantify disaster-related health risks, while studies from Pakistan and other resource-limited settings highlighted fundamental deficits in infrastructure, staffing, and financing that constrained emergency response capacity.^25^ These disparities emphasize the urgency of prioritizing adaptive capacity building in settings where baseline resources are lowest, and climate vulnerability is greatest.

Operational adaptations, defined as changes in the ED workflows, staffing models, or care delivery processes implemented during climate-related disasters, were also described. During Hurricane Harvey, despite a reported 74% loss of inpatient bed capacity, median ED length of stay decreased for both admitted patients and discharges, reflecting changes in care delivery processes implemented during crisis response rather than evidence of causal improvement attributable to specific interventions.^30^ Importantly, the study did not report quantitative staffing increases or workforce expansion; observed differences were attributed to process-level adaptations in patient flow and care delivery under disaster conditions rather than to increased staffing. While these findings suggest that disaster conditions can catalyze innovation, they should not obscure the profound human, operational, and economic costs associated with climate-driven disasters.

Despite these observations, important limitations persist. Most disaster-focused studies examined single hazards, lacked integration with climate projection models, and did not account for compound or cascading events that may pose the greatest risks to emergency care systems. At the policy level, frameworks such as the Sendai Framework for Disaster Risk Reduction emphasize the role of health systems in disaster preparedness and resilience, reflecting global recognition of healthcare as a core component of disaster risk production. However, substantial gaps remain between policy commitments and on-the-ground implementation.^42^

Together, these findings underscore the urgent need for proactive, climate-informed disaster preparedness and align with the shared research and policy priorities detailed in the Common Gaps and Priorities section.

### Toxicology

Our review identified two studies directly linking climate-related exposures—heat and ozone—to ED visits for drug-related toxicity and asthma. These studies underscore the emergency department’s critical role as a frontline detector for emerging climate-sensitive toxicologic harms, while also illustrating the limitations of existing evidence. Traditional toxicology frameworks, which often examine single chemicals under static conditions, may not adequately capture the dynamic, interacting exposures now emerging under climate change^43^,^44^

### Social Vulnerabilities and Mental Health

This review adds to the growing evidence that climate change directly and indirectly worsens mental health, increasing demand on EDs. Consistent with existing literature, we found that rising temperatures and heatwaves were strongly associated with higher ED use for a range of mental health disorders, including schizophrenia, depression, bipolar disorder, substance misuse, and personality disorders. These findings align with previous work showing that both acute climate-related events and chronic environmental stressors can trigger or exacerbate mental illness.^45^–^47^ Importantly, our review also highlighted the disproportionate burden, manifested as higher ED use, among socially and economically marginalized groups, including individuals reliant on public healthcare systems and those experiencing homelessness, reinforcing how climate change amplifies existing inequities.^46^

While the studies reviewed focused primarily on heat exposure, broader literature documents indirect pathways through which climate hazards affect mental health, including trauma related to bushfires, flooding, displacement, and livelihood loss, contributing to post-traumatic stress disorder, depression, anxiety, and increased risk of substance misuse and violence.^46^,^48^ Air pollution has also emerged as an important contributor, with short-term exposures to fine particulate matter and nitrogen dioxide associated with increased psychiatric hospital admissions for depression, schizophrenia, and bipolar disorder in older adults.^33^ Together, these findings underscore the multifaceted pathways by which climate hazards strain mental health and emergency care systems.

Our focus on ED presentations excluded community-based or subclinical mental health impacts and did not systematically capture studies addressing indirect climate pathways. These limitations, along with the need for improved surveillance and integration of mental health with climate resilience planning, are expanded in the Common Gaps and Priorities section, which outlines potential directions for actionable next steps.

### Common Gaps and Priorities

Consistent with the stated objective of this scoping review to identify knowledge gaps across EM subspecialties, we synthesized cross-cutting gaps and priorities that emerged across EMS, trauma, disaster medicine, toxicology, and mental health. This section reflects author synthesis informed by the reviewed literature, rather than findings abstracted directly from individual studies, and is intended to highlight shared vulnerabilities and strategic directions for future research and practice.

#### Limited Geographic and Socioeconomic Scope

Across all domains, the geographic and socioeconomic scope of existing evidence remains limited. Most studies were conducted in high-income countries with well-established emergency care systems, while regions experiencing the greatest climate vulnerability and resource constraints—particularly low- and middle-income countries—were under-represented. This imbalance restricts generalizability and limits insight into settings where climate-related health impacts may be most severe.

#### Methodological Constraints

Methodological limitations were pervasive. The literature was dominated by ecological and retrospective observational designs, with limited use of individual-level data, prospective approaches, or integration of climate projection models. As a result, causal inference remains limited, and the ability to anticipate future emergency care demand under evolving climate scenarios is underdeveloped.

#### Narrow Hazard Focus

A narrow focus on temperature-related exposures further constrained the evidence base. While heat represents a critical and well-characterized hazard, substantially fewer studies examined other climate-related threats, including wildfire smoke, flooding, extreme precipitation, and compound or cascading events. This emphasis may underestimate the full spectrum of climate-sensitive risks facing emergency care systems.

#### Translational Gaps

Despite increasingly consistent documentation of climate-related surges in emergency care utilization, few studies evaluated adaptive interventions. Early warning systems, workforce adaptation strategies, resilient infrastructure design, and community-based prevention efforts were rarely assessed, leaving a persistent translational gap between epidemiologic evidence and actionable preparedness.

#### Equity and Health-System Resilience

Equity considerations emerged as a central but incompletely addressed theme. Populations experiencing homelessness, rural communities, individuals with mental health or substance use disorders, those dependent on under-resourced health systems were consistently identified as vulnerable, yet few studies examined how structural inequities shape exposure, access to care, or adaptive capacity. Addressing these disparities will require research and policy efforts that center equity and capacity building in high-risk, low-resource settings.

Taken together, these gaps highlight several priorities for EM. These priorities reflect our interpretation informed by the reviewed literature and are intended to guide future research and preparedness efforts rather than represent evaluated interventions. Key priorities include the following:

1. Establishing international, multisite surveillance systems capable of detecting emerging hazards in real time
2. Integrating climate projection models directly with health service planning to guide infrastructure investment and workforce allocation
3. Designing and rigorously evaluating targeted interventions, such as climate-triggered EMS dispatch algorithms, heat-resilient power and water systems, and mental health integration into disaster planning, to enhance system resilience
4. Prioritizing capacity building and policy development in the low-resource, high-risk settings

## LIMITATIONS

This review has several limitations. Most included studies focused on temperature-related exposures, with far fewer examining hazards such as wildfire smoke, flooding, or compound climate events. Evidence was also uneven across subspecialties: EMS, trauma, and mental health were relatively well represented, whereas toxicology and certain disaster domains had limited coverage. Further, most included studies were ecological or observational, limiting causal inference and leaving individual-level factors, such as socioeconomic status and comorbidities, insufficiently addressed. Few incorporated climate projection models, restricting insights into future emergency care demand under evolving climate scenarios. Research was also concentrated in high-income, data-rich settings, limiting applicability to low- and middle-income regions where climate impacts may be greatest and emergency care capacity most limited.

Finally, heterogeneity in definitions of climate exposures and emergency care outcomes reduced comparability across studies. While some studies highlighted disparities affecting vulnerable populations, few systematically assessed equity, limiting the ability of this review to fully characterize differential impacts across social and demographic groups.

## CONCLUSION

Climate change is reshaping the landscape of emergency medicine across multiple subspecialties, driving predictable surges in demand, revealing systemic vulnerabilities, and amplifying inequities. Evidence from EMS, trauma, disaster medicine, toxicology, and mental health demonstrates that rising temperatures and extreme weather events are already straining frontline care. However, significant gaps remain, particularly regarding wildfire smoke, flooding, compound hazards, and toxicologic exposures.

Current emergency care frameworks are insufficient for the multi-hazard realities of a warming world. Adaptation will require shifting from reactive, event-based responses toward proactive, climate-informed preparedness. Key priorities include improved surveillance, resilient infrastructure and workforce capacity, and the integration of equity and mental health considerations. Emergency medicine stands at a critical inflection point. By embracing climate resilience as a core competency, the specialty can mitigate immediate risks, safeguard vulnerable populations, and contribute to the development of sustainable, adaptive health systems for the future.

## Figures and Tables

**Table 1:** Characteristics of a scoping review conducted to characterize existing literature at the intersection of climate change and emergency medicine.

Subspecialty	# Studies	Study locations	Study designs/methods
EMS	13	Asia (7), Europe (3), Australia (2), Multinational (1)	Retrospective ecological/observational (4), Time-series (3), Case–crossover (2), Delphi (2), Prognostic (1), Qualitative (1)
Trauma	4	China (2), Korea (1), Pakistan (1)	Time series (3), Policy analysis (1)
Disaster	7	China, Pakistan, Taiwan, Ireland, USA	Observational (5), Policy analyses (2)
Toxicology	2	USA (California, nationwide projection)	Case-crossover observational (1), Modeling (1)
Mental Health	9	Brazil, Beijing, Toronto, USA, Canada, Sydney	Observational (9)

*EMS*, emergency medical services; *USA*, United States of America.

**Table 2:** Climate vulnerabilities and policy implications identified in a scoping review conducted to characterize existing literature at the intersection of climate change and emergency medicine.

Subspecialty	Climate Vulnerable Populations	Policy/Practice Gaps
EMS	Males, working-age adults (18–44), elderly ≥ 65; disparities between income-level countries	Lack of EMS adaptation planning; disparities in resources between high- and low-income regions; limited system-level strategies for compound climate events
Trauma	Males, adults 18–59, elderly ≥ 65; lag effects 1–6 days	No training/educational interventions identified; inadequate trauma system preparedness in climate-vulnerable regions (e.g., Pakistan); translational gap from data to protocols
Disaster	Nurses with low preparedness (74% lacked knowledge); rural vs urban disparities; varied event patterns by disaster type	Critical gaps in disaster preparedness infrastructure; limited hospital emergency management offices; need for structured training programs and resilient infrastructure
Toxicology	Individuals with substance use disorders (amphetamine, cocaine, opioids); asthma patients in high-ozone areas	Overdose risks exacerbated by rising temperatures; need for integrated heat- and climate-adapted substance use interventions; importance of climate mitigation for respiratory outcomes
Mental Health	Women, youth, psychotic and substance-use disorders, homeless, low SES, dementia patients	Inadequate integration of climate risk into mental health systems; lack of preventive strategies for vulnerable groups; need for heat-adaptive services and early-warning interventions

*EMS*, emergency medical services; *SES*, socioeconomic status.
